# Stability of the Resistance to the Thiosemicarbazone Derived from 5,6-Dimethoxy-1-Indanone, a Non-Nucleoside Polymerase Inhibitor of Bovine Viral Diarrhea Virus

**DOI:** 10.1371/journal.pone.0100528

**Published:** 2014-06-20

**Authors:** Eliana F. Castro, Rodolfo H. Campos, Lucía V. Cavallaro

**Affiliations:** Cátedra de Virología, Facultad de Farmacia y Bioquímica, Universidad de Buenos Aires, Buenos Aires, Argentina; University of Alberta, Canada

## Abstract

Bovine viral diarrhea virus (BVDV) is the prototype *Pestivirus*. BVDV infection is distributed worldwide and causes serious problems for the livestock industry. The thiosemicarbazone of 5,6-dimethoxy-1-indanone (TSC) is a non-nucleoside polymerase inhibitor (NNI) of BVDV. All TSC-resistant BVDV variants (BVDV-TSC^r^ T_1–5_) present an N264D mutation in the NS5B gene (RdRp) whereas the variant BVDV-TSC^r^ T_1_ also presents an NS5B A392E mutation. In the present study, we carried out twenty passages of BVDV-TSC^r^ T_1–5_ in MDBK cells in the absence of TSC to evaluate the stability of the resistance. The viral populations obtained (BVDV R_1–5_) remained resistant to the antiviral compound and conserved the mutations in NS5B associated with this phenotype. Along the passages, BVDV R_2_, R_3_ and R_5_ presented a delay in the production of cytopathic effect that correlated with a decrease in cell apoptosis and intracellular accumulation of viral RNA. The complete genome sequences that encode for NS2 to NS5B, Npro and Erns were analyzed. Additional mutations were detected in the NS5B of BVDV R_1_, R_3_ and R_4_. In both BVDV R_2_ and R_3,_ most of the mutations found were localized in NS5A, whereas in BVDV R_5_, the only mutation fixed was NS5A V177A. These results suggest that mutations in NS5A could alter BVDV cytopathogenicity. In conclusion, the stability of the resistance to TSC may be due to the fixation of different compensatory mutations in each BVDV-TSC^r^. During their replication in a TSC-free medium, some virus populations presented a kind of interaction with the host cell that resembled a persistent infection: decreased cytopathogenicity and viral genome synthesis. This is the first report on the stability of antiviral resistance and on the evolution of NNI-resistant BVDV variants. The results obtained for BVDV-TSC^r^ could also be applied for other NNIs.

## Introduction

Bovine viral diarrhea virus (BVDV), along with classical swine fever virus and border disease virus, is a member of the genus *Pestivirus*. This genus belongs to the family *Flaviviridae*, which also includes the genera *Hepacivirus* (hepatitis C virus) and *Flavivirus* (yellow fever virus, Dengue fever virus and West Nile virus).

BVDV infection is distributed worldwide and tends to be endemic in many countries [1], [2]. In Argentina, the prevalence of BVDV antibodies in adult cattle is around 70% [3], [4]. BVDV infections lead to a reduction in milk production, lower conception rates, respiratory disorders and death of the animals that acquire the acute infection, thus causing serious economic losses for the livestock industry. Moreover, fetal infection leads to persistently infected cattle, which are generally smaller, are more susceptible to other infections and eventually develop a lethal mucosal disease.

BVDV is an enveloped single-stranded (+) RNA virus. Its genomic RNA is about 12.5 kb long and consists of a single open reading frame (ORF) flanked by 5′ and 3′ non-translated regions (NTRs). The ORF encodes a polyprotein of approximately 3,900 amino acids that is co- and post-translationally processed to mature viral proteins by cellular and viral proteases. The viral proteins are sequentially designated Npro, C, Erns, E1, E2, p7, NS2, NS3, NS4A, NS4B, NS5A and NS5B. The autoprotease Npro is a non-structural (NS) protein that plays a role in blocking IFN-α/β induction [5]–[8]. The capsid protein (C) is followed by three virion glycoproteins: Erns, E1, and E2. Erns encodes an RNase that is secreted in nonvirion forms [9] and targets extracellular RNA, a major viral signal that triggers IFN synthesis [10], [11]. The rest of the polyprotein is processed to NS proteins, of which only NS3 through NS5B are required for RNA replication [12], [13]. NS2, together with the amino terminus of NS3, functions as an autoprotease that cleaves the NS2-NS3 junction of the polyprotein. This cleavage is required for RNA replication and is linked to BVDV cytopathogenicity and pathogenesis [14], [15]. NS3 is a multifunctional protein with a helicase/nucleoside triphosphatase and serine protease activity responsible for all downstream polyprotein cleavages [16]–[19]. NS4A is tightly associated with NS3 and serves both as a cofactor for NS3 protease activity and as a tether that localizes NS3 to membranes [17], [20]. NS4B is believed to function as an integral membrane scaffold upon which the replicase complex (RC) assembles [21]. NS5A is a phosphoprotein that plays an essential role in BVDV replication and pathogenesis [22]–[24]. Finally, NS5B is the viral RNA-dependent RNA polymerase that catalyzes viral RNA synthesis [25]–[29].

BVDV isolates are categorized into two biotypes according to their effect on cultured cells: noncytopathic (ncp) isolates, which infect permissive host cells without causing cell death, and cytopathic (cp) isolates, which produce rapid cytopathic effects (CPE) and kill cells [30]. Only ncp isolates are able to establish the persistent infection. This difference is associated with distinct interactions between each biotype and the host innate immune response against the viral infection, which is active early during gestation. Cp biotypes emerge from ncp biotypes exclusively in persistently infected animals and are invariably associated with the mucosal disease [12], [31]. The induction of apoptosis plays a significant role in the pathology of the cp isolates both in infected cell cultures and in the clinical manifestations of the mucosal disease [32]–[35].

The thiosemicarbazone of 5,6-dimethoxy-1-indanone (TSC) is a non-nucleoside polymerase inhibitor of BVDV (EC_50_ 1.8±0.6.µM) [36]–[38]. In a previous study, we selected five independent populations of TSC-resistant BVDV (BVDV-TSC^r^; T_1–5_) (TSC EC_50_>80.0 µM). All of them carry an NS5B N264D mutation, whereas BVDV-TSC^r^ T_1_ also shows an NS5B A392E mutation [38].

Studies on the resistance to an antiviral agent are highly important for the development and therapeutic application of such antiviral agent. Given that the impact of resistance is difficult to predict, it is important to evaluate not only the emergence of resistance but also its stability and the effect of the associated mutations on the viral replicative fitness in an antiviral-free environment. Therefore, the aim of the present work was to evaluate the stability of the resistance to TSC. To this end, we carried out 20 passages of BVDV-TSC^r^ T_1–5_ in the absence of TSC. We also describe the molecular and biological characterization of the viral populations obtained, in terms of infectious virus production and cytopathogenesis.

## Materials and Methods

### Cells and virus

Madin Darby bovine kidney cells (MDBK NBL-1; ATCC CCL-22) were grown in Eagle's Minimal Essential Medium (EMEM), supplemented with 10% γ irradiated fetal bovine serum (FBS, PAA Laboratories, Pasching, Austria) (growing medium). BVDV type 1 NADL strain, cytopathic biotype (BVDV-1, ATCC VR 534) was provided by Dr Laura Weber, INTA Castelar, Argentina. Wild type (wt) BVDV p0 was obtained after three successive steps of biological cloning of BVDV NADL in MDBK cells. TSC-resistant BVDV (BVDV-TSC^r^ T_1–5_) were obtained from wt BVDV p0 after ten passages in MDBK cells with increasing concentrations of TSC [38].

### Passages of BVDV-TSC^r^ in the absence of TSC

MDBK cells were infected with BVDV-TSC^r^ T_1–5_ at a multiplicity of infection (MOI) of 0.01. Cell cultures were incubated in infection medium (infection medium: EMEM supplemented with 2.5% Donor Horse Serum -DHS- Gibco) in a 5% CO_2_ incubator at 37°C until cell monolayers presented 80–90% of viral CPE. The supernatant was collected and clarified by centrifugation for 10 min at 1,000×g at 4°C. After titration of the virus in MDBK cells, the virus was used to infect new cell monolayers to generate the next passage. This procedure was repeated twenty times, and the virus obtained from each BVDV-TSC^r^ T_1–5_ was named BVDV R_1–5_.

### Plaque reduction assay for BVDV

To evaluate the antiviral activity of TSC against BVDV R_1–5_, MDBK cells were seeded in growing medium at a density of 1.3×10^5^ cells per well in a 24-well plate and incubated for 24 h at 37°C in a 5% CO_2_ incubator. Then, cells were infected with approximately 100 plaque-forming units (PFUs) of BVDV R_1–5_ or wt BVDV p0. Following 1 h of adsorption at 37°C, the inoculum was removed, cell monolayers were washed twice with phosphate-buffered saline (PBS), and 1 ml of overlay medium (2.5% DHS and 0.8% methylcellulose) containing 80 µM of TSC was added in each well. Mock-infected cells with and without TSC, and infected cells without TSC were included as controls. After 72 h of incubation at 37°C with 5% CO_2_, cells were fixed with formalin 10%, stained with 0.75% crystal violet, and the viral plaques counted.

### Apoptosis measurements

MDBK cells were seeded in a 24-well plate as described above. After 24 h in a 5% CO_2_ incubator at 37°C, cells were infected with wt BVDV p0 or R_1–5_ (MOI 0.01). Following 1 h of adsorption at 37°C, the inoculum was removed, cell monolayers were washed twice with PBS and 1 ml of infection medium was added in each well. Mock-infected cells were included as controls. After 48 h of incubation at 37°C with 5% CO_2_, apoptosis was measured in infected and mock-infected cell cultures.

For apoptosis measurements, PE Annexin V Apoptosis Detection Kit I (BD Pharmigen, NJ, USA) was used following the manufacturer's instructions. Briefly, supernatants from infected and mock-infected cells were harvested and cells in the monolayer were collected by trypsinization and resuspended in 0.5 ml of infection medium. Adherent and floating cells were pooled and pelleted by centrifugation at 500×g for 10 min. Pelleted cells were washed twice with 0.5 ml of cold PBS. Before the second centrifugation (500×g, 10 min), cells were counted in a Neubauer chamber. To obtain the dead-cell control for apoptosis measurements, one mock-infected cell suspension was heated at 65°C for 30 min. Then, cells were resuspended in binding buffer (4×10^6^ cells/ml) and 100 µl of each cell suspension was stained with 5 µl Annexin V (conjugated with Phycoerythrin- PE-) and 5 µl 7-Amino-Actinomycin (7AAD) and incubated for 15 min at room temperature in the dark. Finally, 400 µl of binding buffer was added and the cells were analyzed by flow cytometry (PARTEC, PAS-III). PE Annexin V and 7AAD fluorescence was collected at FL 2 (550–600 nm) and FL4 (640–700 nm) channels, respectively. Histograms and density plots of 20,000 events were obtained and analyzed with WinMDI 2.9 software [39].

At 48 h p.i. some infected cultures already presented viral CPE, which is caused by cell apoptosis. The number of cells that would have died by this mechanism and could no longer be detected was estimated by the difference between the number of cells counted in a Neubauer chamber in the mock-infected cultures (MIC) and those counted in the infected ones (IC) (*A:* *N* *° undetected dead cells  =  *N*° cells _MIC_ − *N*° cells _IC_*). Then, the number of apoptotic cells in each infection (i.e. in the remaining adherent and floating cells) was quantified by AnV binding and flow cytometry as stated above (*B: *N*° apoptotic cells  =  %* AnV_pos_ *× *N*° cells _IC_/100*). Finally, the total cells that would die by each viral infection was estimated as *% dead cells (normalized to uninfected)  =  (A+B) × 100/*N*° cells _MIC_*.

### Multiple-step growth curve

To quantify the infectious virus produced by BVDV R_1–5_ in the absence of TSC, MDBK cells were seeded in a 24-well plate as stated above and infected with BVDV R_1–5_ or wt BVDV p0 (MOI 0.01). Following 1 h of adsorption at 37°C, the inoculum was removed, cell monolayers were washed twice with PBS, and 1 ml of infection medium was added to each well. The culture supernatant was collected every 24 h for 7 days and clarified by centrifugation for 10 min at 1,000×g at 4°C. The infectious virus in each sample was quantified by the PFU method in MDBK cells.

Given that BVDV R_4_ and R_5_ showed CPE and levels of cell apoptosis similar to those of BVDV R_1_ and R_2_, respectively (see results section), they were not included in the following assays (unless otherwise indicated).

### Fluorescent microscopy

MDBK cells were seeded in a 24-well plate at a density of 1.3 ×10^5^ cells per well containing a circular slide cover of 10 mm in diameter. After 24 h in a 5% CO_2_ incubator at 37°C, cells were infected with BVDV R_1_, R_2,_ R_3_ or wt BVDV p0 (MOI 0.01). Following 1 h at 37°C, the inoculum was removed, cell monolayers were washed twice with PBS, and 1 ml of infection medium was added to each well. At 24, 48 and 72 h p.i., the cell culture medium was removed and cell monolayers were fixed with 4% formaldehyde/PBS for 15 min at room temperature. After washing three times with PBS, cultures were incubated with 0.5 ml of blocking buffer (10% Bovine seralbumin -BSA-; 0.3% Triton X-100 in PBS) for 30 min. Then, slide covers were removed and incubated overnight at 4°C with 30 µl of anti-BVDV NS3 Monoclonal Antibody (diluted 1∶1000 in PBS 0.1% Triton X-100; 1% BSA), kindly provided by Dr Alejandra Capozzo (Instituto de Virología, INTA, Castelar, Buenos Aires, Argentina). Slide covers were washed three times with PBS and incubated protected from light for 2 h at room temperature with Alexa Fluor 488 Goat Anti-Mouse antibody (Invitrogen) (diluted 1∶1000 in PBS 0.1% Triton X-100; 1% BSA). Afterwards, cells were washed three times with PBS and the nucleus stained with 300 µl of DAPI (Invitrogen) (dilution 1∶100) for 5 min. The slides were mounted with FluorSave (Calbiochem) and the cells were observed with a Nikon Eclipse 80i microscope with a 20× objective (numerical aperture 0.40). The images were captured with a digital camera (Nikon Digital Sight DS-Qi1Mc) and processed using ImageJ software.

### Viral RNA production

MDBK cells were seeded in a 24-well plate as stated above. After 24 h in a 5% CO_2_ incubator at 37°C, cells were infected with BVDV R_1_, R_2,_ R_3_ or wt BVDV p0 (MOI 1). Following 1 h of adsorption on ice, the inoculum was removed, cell monolayers were washed twice with PBS, and 1 ml of infection medium was added to each well. After 24 h of incubation at 37°C, intracellular RNA was extracted with TRIzol reagent (Gibco-BRL, CA, USA). RNA was retrotranscribed with random hexamers (Biodynamics) at 37°C for 90 min, using 0.5 U/μl M-MLV reverse transcriptase (Promega, Madison, WI, USA), and quantified by Real-time PCR.

### Real-time PCR

To measure the intracellular viral RNA (ivRNA), a 108-bp fragment from the 5′NTR was amplified with the following oligonucleotides: 5′NTR forward 5′GAGGCTAGCCATGCCCTTAGT3′ and 5′NTR reverse 5′TCGAACCACTGACGACTACCCT3′. The reaction was carried out in 2X Power SYBR Green PCR Master Mix (Applied Biosystems, UK) in an ABI 7500 apparatus (Applied Biosystems), using the following experimental run protocol: 10 min of activation at 95°C, followed by 45 cycles of amplification and quantification (15 sec at 95.0°C, 1 min at 60.0°C and 35 sec at 78.5°C) during which the SYBR GREEN signal was measured. To normalize target amplification data, a 91-bp fragment of bovine β-actin mRNA was amplified simultaneously in each sample and used as endogenous control (forward oligonucleotide: 5′CCCACACGGTGCCCATCTAT3′ and reverse oligonucleotide: 5′ CCACGCTCCGTGAGGATCTTC3′). Each sample was tested in triplicate and both positive and negative controls were included.

Data were analyzed by means of the relative standard curve method [40] to quantify the ivRNA at 24 h p.i. in the cell cultures infected with BVDV R_1_, R_2_ and R_3_ in relation with those infected with wt BVDV p0.

### Sequencing

Total RNA was extracted with TRIzol reagent (Gibco-BRL) from wt BVDV p0, BVDV-TSC^r^ T_1–5_ and BVDV R_1–5_ virus suspensions obtained from clarified supernatants of infected cells. The RNA obtained was denatured at 70°C for 5 min and primed with the designed specific oligonucleotides (Table 1). The reverse transcription reaction was performed at 42°C for 90 min, using 10 U/μl of ArrayScript™ Reverse Transcriptase (Ambion, Inc.). The PCR fragments that cover the complete coding region of all the non-structural proteins (Npro, NS2-NS5B) and the glycoprotein Erns were obtained using specific oligonucleotides (Table 1) by PCR amplification with 0.025 U/μl of PFU DNA polymerase (Promega). Both DNA strands were sequenced using an ABI ABI3130XL sequencer 131 (Applied Biosystems; Unidad de Genómica, Instituto de Biotecnología, INTA, Castelar, Buenos Aires, Argentina). The sequences were aligned using ClustalX 1.83 [41], edited with BioEdit v 7.0.9.0 [42] and compared with the BVDV strain NADL (p0 and GenBank accession no. M31182.1; AJI33738.1; NP_776270.1; NP_776271.1; NC_001461.1).

**Table 1:** Oligonucleotides used for retrotranscription (RT) reactions and PCR amplifications.

Primer	Sequence (5′-3′)	Position in the genome
Npro region		
PR S1	GCCCACTGTATTGCTAC	344–360
PR AS1	CCCCTTTCTAGTCTGTCG	937–954
Erns region		
PR S2	AGGAATCACGCAAGAAACTG	1,116–1,135
PR AS2	GTCACTAGCCCTATCATACC	1,539–1,558
PR S3	TTGAACCCTGGATTCTAGTC	1,455–1,474
PR AS3	AATTTCCCAGGGCCGAC	1,970–1,986
NS2-NS3 region		
RT1	CTTCCCCTTCCTTATAGAGAG	8,261–8,281
PR S4	AATACTGGTTTGACCTGGAG	3,435–3,454
PR AS4	CGCCAGTTCTTTCAGTTCAGT	7,646–7,666
SR S5	CGCTGAGTCCATATTAGTGGT	3,481–3,501
SR AS5	ACTGAACTTATCCCGCCT	5,605–5,622
SR S6	CTGCCGTGTGTAAGAAGAT	5,427–5,445
SR AS6	GTCTTCTAGTCTCTGGTCCT	7,575–7,594
NS4A-NS4B region		
RT2	GTAGTGTTAGCTTGAGGTAGATA	12,477–12,498
PR S7	TAGACTGGCCTGATCCTG	7,401–7,418
PR AS7	GTTGGCAATCTGATCCTCT	8,826–8,844
NS5A-NS5B region		
RT2	GTAGTGTTAGCTTGAGGTAGATA	12,477–12,498
PR S8	CTGTATGGGGTTTACTACAAAGG	8,567–8,589
PR AS8	TCTTCTTGAGGTGGACGG	12,417–12,434
SR S9	CTGTATGGGGTTTACTACAAAGG	8,567–8,589
SR AS9	TTGGTAAGCTGATGCCATGTG	10,295–10,315
SR S10	AGGAGATGTTGGAGAGGTAA	10,090–10,109
SR AS10	TCTTCTTGAGGTGGACGG	12,417–12,434

Sequences and positions in the genome of BVDV NADL of the oligonucleotides designed and used for RT reactions and PCR amplifications of Npro, glycoprotein Erns and non-structural proteins coding region. Positions in the BVDV genome are according to BVDV NADL genome sequence GenBank accession no AJ133738.1.

### Statistical analysis

The results were analyzed by Student's t test using InfoStat version 2011 [43]. A statistically significant difference was considered if ***p*** values were less than 0.050.

## Results

### The resistance to TSC is conserved after 20 passages

**Figure 1:**
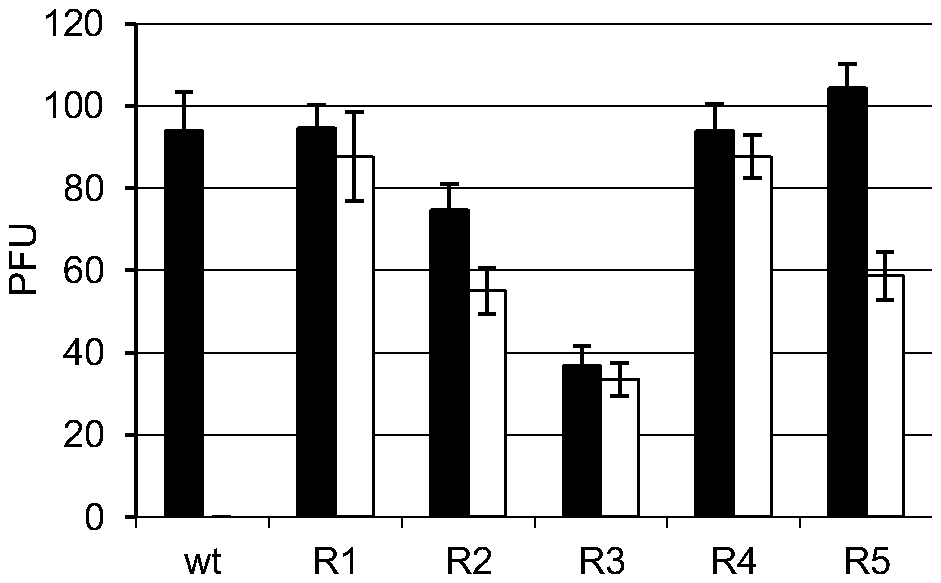
Antiviral activity of TSC against BVDV R_1–5_. BVDV-TSC^r^ T_1–5_ were propagated in MDBK cells in the absence of TSC during 20 passages. The antiviral activity of 80 µM of TSC against the viral populations obtained (BVDV R_1_–R_5_) and wt BVDV p0 was evaluated by plaque reduction assays. White and black bars indicate the number of viral plaque forming units (PFUs) formed in the presence or in the absence of TSC, respectively. (TSC vs untreated: R_1_ ***p*** =  0.560; R_2_ ***p*** = 0.054; R_3_ ***p*** =  0.449; R_4_ ***p*** =  0.382; R_5_ ***p*** =  0.002; p0 ***p*** =  0.003).

BVDV R_1–5_ were obtained after 20 passages of BVDV-TSC^r^ T_1–5_ in the absence of TSC. BVDV R_1–4_ were not significantly inhibited by TSC, whereas wt BVDV p0 was completely inhibited (TSC vs untreated: R_1_ ***p*** =  0.560; R_2_ ***p*** =  0.054; R_3_ ***p*** =  0.449; R_4_ ***p*** = 0.382; p0 ***p*** =  0.003) (Figure 1). However, with the number of PFUs analyzed, BVDV R_5_ showed a reduction in the number of viral plaques of approximately 44%, suggesting a slight decrease in drug resistance (TSC vs untreated: R_5_ ***p*** =  0.002).

### A delay in CPE production is observed in some viral populations during passages

The infection of MDBK cells with wt BVDV NADL cp at a MOI of 0.01 required 48 h of incubation at 37°C to reach 80–90% of CPE in the cell monolayer. However, along the passages of BVDV-TSC^r^ T_1–5_ in the same conditions, some viral populations showed a delay in producing CPE. To reach 80–90% of CPE, the infections with BVDV-TSC^r^ T_2_ and T_5_ required 72 h of incubation from the 1^st^ to the 3^rd^ passage and then 120 h of incubation from the 4^th^ to the 20^th^ passage. Similarly, BVDV-TSC^r^ T_3_ required 120 h of incubation from the 13^th^ to the 20^th^ passage. This delay in producing CPE was also observed in the growth of viral plaques when the infected cultures were incubated in semi-solid medium. BVDV R_2_, R_3_, and R_5_ needed 72–96 additional hours of incubation to properly exhibit the viral plaques.

### The delay in producing CPE is correlated with a reduced induction of apoptosis in MDBK cells

**Figure 2:**
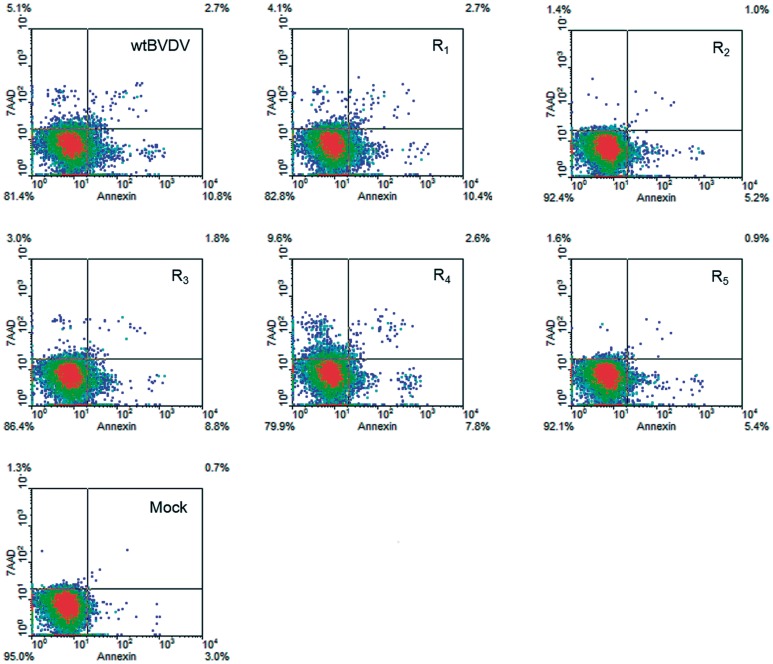
Apoptosis measurements. MDBK cells were infected with wt BVDV p0 or BVDV R_1–5_ (MOI 0.01) and at 48 h p.i. apoptotic cells were measured by incorporation of PE Annexin V and 7AAD dyes and analyzed by flow cytometry. Mock-infected cultures were added as control. Representative density plots (20,000 events) obtained with WinMDI software are displayed. Quadrants delimitating positive (pos) and negative (neg) events are shown and the percentage of events in each quadrant is indicated. The populations of PE Annexin V−/7AAD−, PE Annexin V+/7AAD− and PE Annexin V+/7AAD+ correspond to viable cells, early apoptotic cells, and late apoptotic cells.

BVDV cp isolates kill cells by triggering apoptosis [30], [32], [44]. In accordance with the microscopic observations of the viral CPE, the infection of MDBK cells with BVDV R_1_ or R_4_ produced levels of apoptosis similar to those produced by wt BVDV p0 (13.1%, 10.8%, and 11.0% of PE Annexin-positive cells, respectively), whereas the infections with BVDV R_2_ and R_5_ produced lower level of apoptosis (5.7% and 5.5% of PE Annexin-positive cells, respectively). BVDV R_3_ showed an intermediate value (9.1% of PE Annexin-positive cells) (Figure 2).

**Figure 3:**
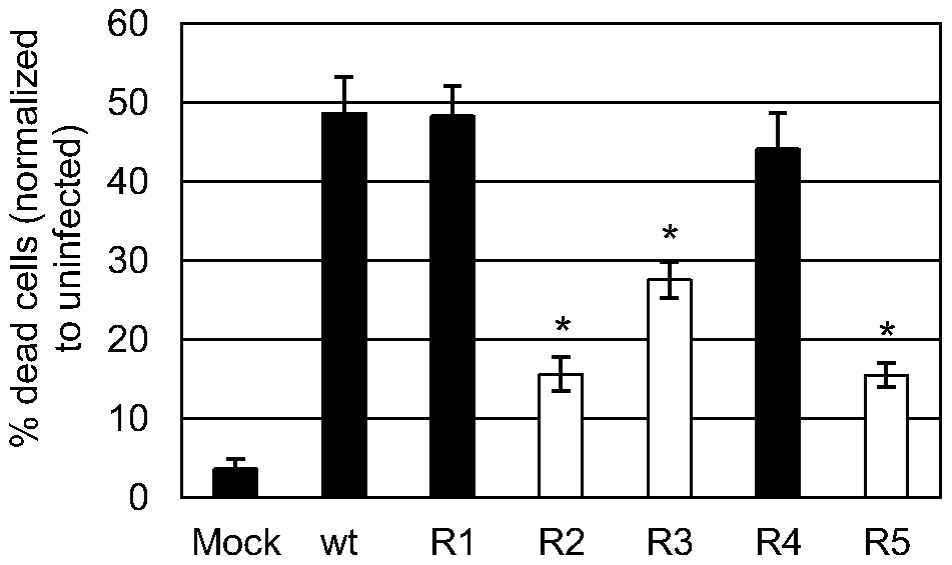
Infection with BVDV R_2_, R_3_ and R_5_ leads to a decrease in dead cells. MDBK cells were infected with wt BVDV p0 or BVDV R_1–5_ (MOI 0.01) and at 48 h p.i. apoptotic cells were measured as stated in Figure 2. Considering the differences in CPE between each viral infection, the total cells that would die by viral CPE was estimated (see Materials and Methods Section). The results are summarized in a bar graph as mean ± SD of % dead cells in relation to the mock-infected culture. *p<0.050; Student's t test vs wt BVDV p0.

Due to viral CPE, the number of remaining cells at 48 h p.i. in the cultures infected with wt BVDV p0, R_1_ or R_4_ was lower than that in the cultures infected with BVDV R_2_, R_3_ or R_5_ (2.4–3.0×10^5^ and 4.8–5.5×10^5^, respectively) and that in mock-infected cultures (6.4×10^5^). Taking into account that apoptotic cells could be quantified only in the remaining adherent and floating cells, the total number of cells that would die by viral CPE in each infection was estimated (see Materials and Methods Section). Having considered the differences in CPE between each viral infection, the results showed that the number of cells that would die by BVDV R_2_, R_3_ and R_5_ infections was markedly lower than that in wt BVDV p0 infection (15.6%, 27.5%, 15.5% and 48.6% of estimated dead cells, respectively) (Figure 3).

### The delay in CPE is not caused by a decreased infectious virus production

**Figure 4:**
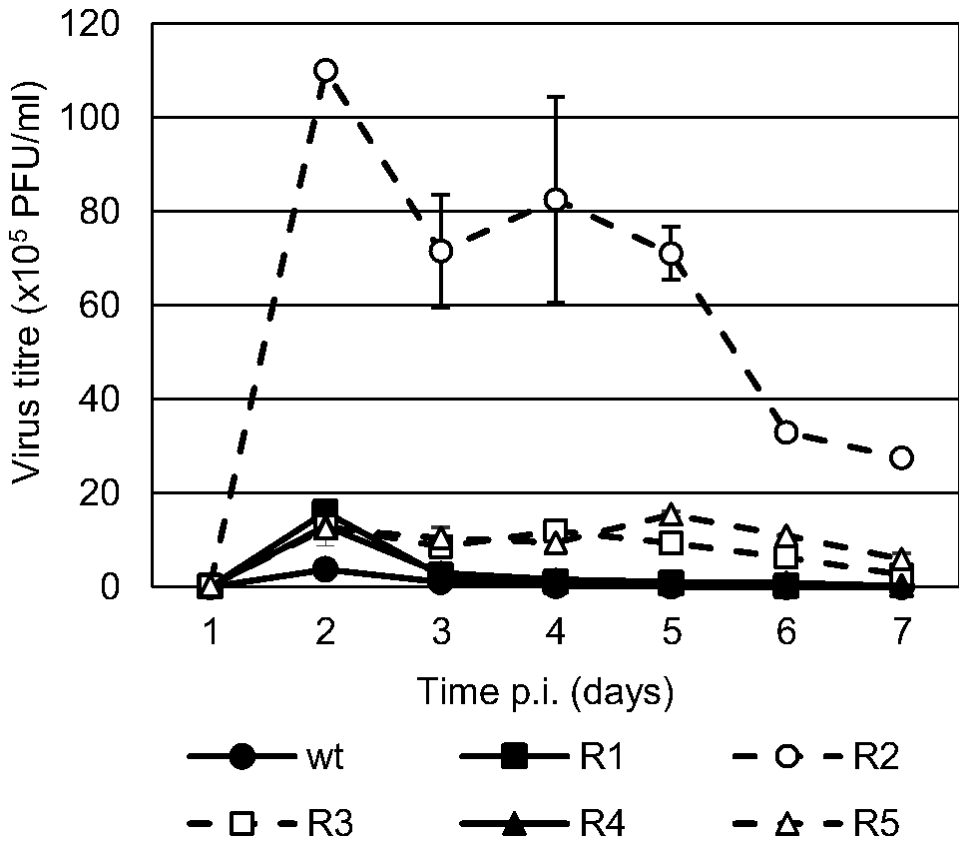
Multiple-step growth curve. MDBK cells were infected with wt BVDV p0 or BVDV R_1–5_ (MOI 0.01). The viral progeny in the culture supernatant was collected every 24 h for 7 days and titrated by PFU. Viruses with altered CPE (BVDV R_2_, R_3_, and R_5_) are displayed in dashed lines.

Although some viral strains produced no CPE, all viral populations showed the highest titers of infectious virus at 48 h p.i. (Figure 4). At that time, most BVDV R (R_1_ and R_3–5_) showed slightly higher virus titers (approximately three times higher) than wt BVDV p0, but, notably, BVDV R_2_ showed viral titers approximately 30 times higher.

On the other hand, wt BVDV p0, R_1_ and R_4_ showed a marked decrease in viral titers at 72 h p.i., consistent with the lack of viable host cells as a result of the viral CPE. However, MDBK cells infected with R_2,_ R_3_ and R_5_ continued producing infectious virus for at least four additional days.

**Figure 5:**
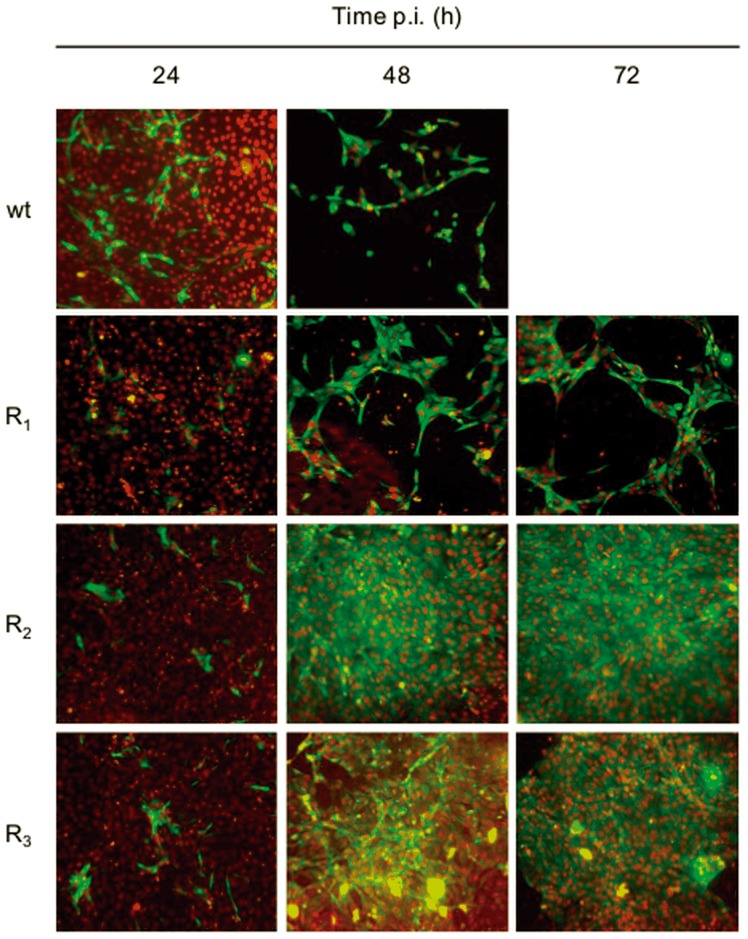
MDBK cells infected with wt BVDV and BVDV R viruses. MDBK cells were infected with wt BVDV p0, BVDV R_1_, R_2,_ or R_3_ (MOI: 0.1) and IF-stained using a monoclonal antibody against the NS3 protein at 24, 48 and 72 h p.i. The nucleus was stained with DAPI. Images were taken with a 20× objective (numerical aperture 0.40) and processed using ImageJ software (the nuclei were pseudocolored with red for better illustration).

In addition, expression of the NS3 antigen was monitored by indirect immunofluorescence. In accordance with the results described above, cell monolayers infected with BVDV R_2_ or R_3_ were still confluent until 72 h p.i and maintained a continuous production of NS3 (Figure 5).

### Viruses that have a delayed CPE exhibit less accumulation of viral RNA

**Figure 6:**
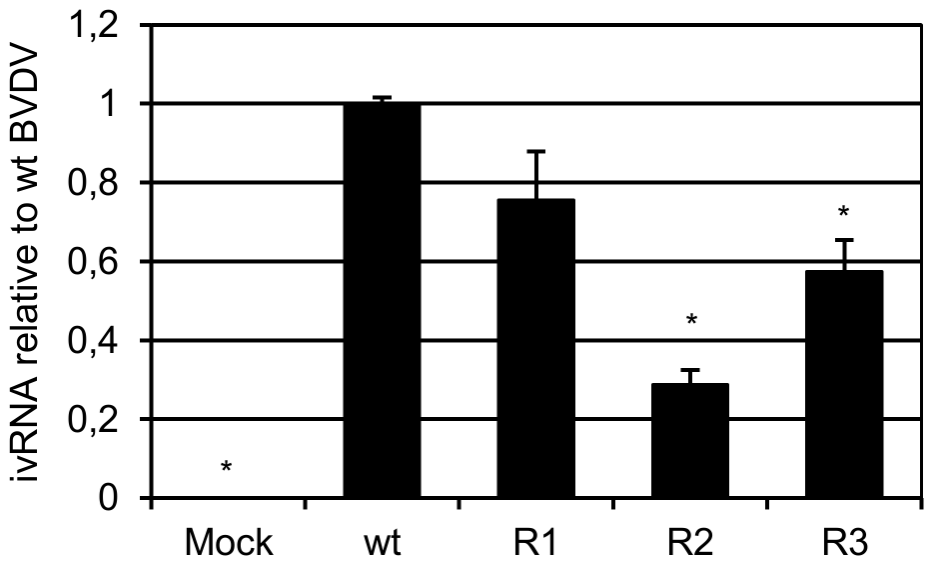
Viral RNA production. MDBK cells were infected with wt BVDV p0, or BVDV R_1_, R_2_ or R_3_ (MOI 1) and the intracellular viral RNA (ivRNA) was quantified at 24 h p.i. by real-time PCR. For each infection with BVDV R viruses, the results are expressed as ivRNA relative to wt BVDV p0. Mock-infected cells were added as control in each experiment. The results are shown as mean ± SD from two independent experiments in triplicate. *p<0.050; Student's t test vs wt BVDV p0.

One of the factors that contribute to cell apoptosis and viral cytopathogenicity is the accumulation of viral RNA in the cells infected with cp BVDV [45]. In line with this, our results showed that the ivRNA levels in BVDV R_2_ and R_3_ infections were lower than those of wt BVDV p0 and R_1_. BVDV R_2_, which together with BVDV R_5_ exhibited the lowest level of cell apoptosis in the infected cultures, also presented the lowest level of ivRNA accumulation (approximately three times lower than wt BVDV p0). On the other hand, BVDV R_3_, which showed an intermediate level of decrease in cell apoptosis, also presented intermediate levels of ivRNA (Figure 6).

### Resistance mutations are conserved and additional substitutions were fixed in BVDV R_1–5_

The nucleotide sequence of Npro, Erns and the complete NS region of wt BVDV p0, BVDV-TSC^r^ T_1–5_ and BVDV R_1–5_ genomes were obtained by direct sequencing (GenBank accession numbers KJ608467-KJ608499).

**Figure 7:**
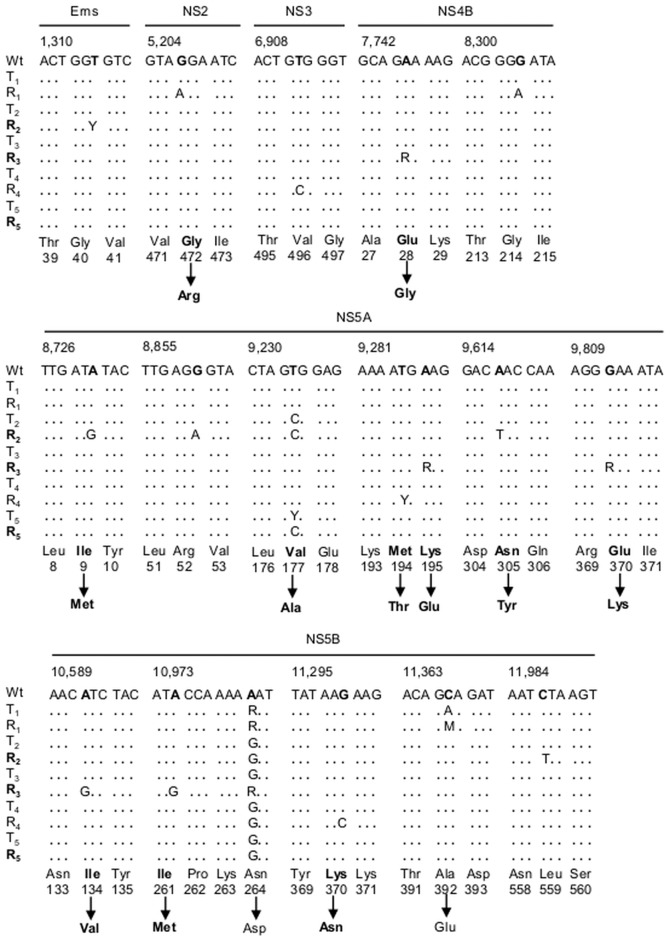
Molecular characterization of BVDV R_1–5_. The complete nucleotide sequence of the Erns, Npro and NS region from wt BVDV p0, BVDV-TSC^r^ T_1–5_ and BVDV R_1–5_ genomes was obtained by direct sequencing. The nucleotide sequences are arranged in three or four codons and the position in the genome of the first nucleotide of each group of sequences is indicated above according to the complete genome of BVDV (GenBank accession no. NC_001461.1). Only the genome regions which showed mutations are displayed. Each BVDV-TSC^r^ T-R pair is shown in gray or white rows and, for non-synonymous mutations, amino acid changes are indicated below the corresponding nucleotide sequence, indicating the number of amino acids for each protein. Viruses that showed altered CPE (BVDV R_2_, R_3_ and R_5_) are indicated in boldface.

After 20 passages without TSC, the nucleotide sequences of the genomic region that codifies the NS5B protein showed that BVDV R_1–5_ conserved the mutations previously associated with the resistance to TSC [38]: A10,982G (N264D) in BVDV R_1–5_ and C11,367A (A392E) in BVDV R_1_ (which showed mixed populations of nucleotides A/G and C/A in positions 10,982 and 11,367, respectively) (Figure 7).

Secondly, several mutations were fixed along the sequences studied: in BVDV R_1_: NS2 G5,207A (G472R); in BVDV R_2_: Erns T1,315Y (Y: mix of T and C), NS5A A8,731G (I9M), NS5A G8,860A, NS5A A9,617T (N305Y), and NS5B C11,987T; in BVDV R_3_: Erns T1,706G (C171G), NS4B A7,745R (R: mix of A and G) (E28G), NS5A A9,287R (K195E), NS5A G9,812R (E370K), NS5B A10,592G (I134V) and A10,975G (I261M); in BVDV R_4_: NS3 T6,912C, NS5A T9,285Y (M94T), NS5B C10,768T and NS5B G11,300C (K370N). Some other mutations (not associated with antiviral resistance) were conserved between BVDV-TSC^r^ T_1–5_ and their respective BVDV R_1–5_: NS5B A10,723G in T_1_-R_1_ and NS5A T9,234C in T_2_-R_2_ (V177A). Notably, no new mutation was detected in BVDV R_5_ after 20 passages. However, whereas BVDV-TSC^r^ T_5_ presented mixed population of T and C in nucleotide T9,234 (NS5A V177), BVDV R_5_ presented only C (A177) (Figure 7). This suggests that this mutation would have become fixed in the viral population during the passages in the absence of TSC.

## Discussion

We have previously reported the selection of TSC-resistant BVDV mutants (BVDV-TSC^r^ T_1–5_) with mutations in NS5B (T_1–5_: N264D; T_1_: A392E) [38]. In the present study, we performed 20 passages of BVDV-TSC^r^ T_1–5_ in the absence of TSC to analyze their genetic stability.

The resistance to TSC remained stable and mutations in NS5B associated with resistance were conserved in all the viral populations obtained (BVDV R_1–5_). However, BVDV R_1–5_ produced higher titers of infectious virus than wt BVDV p0 and, along the passages in the antiviral-free environment, three out of the five viral populations (BVDV R_2_, R_3_ and R_5_) showed a delay in producing CPE.

It is known that drug-resistant mutants may have fitness costs in the absence of the selecting drug, but the occurrence of compensatory mutations may allow them to partially or even completely regain fitness [46]–[48]. A previous work has shown that BVDV variants that carry the NS5B N264D mutation, which confers resistance to TSC and to other non-nucleoside polymerase inhibitors (NNI) [49], [50], present a replication disadvantage when competing with the wild-type (wt) virus in an antiviral-free environment [51]. In the present work, the replication of BVDV-TSC^r^ in the absence of TSC led to the fixation of different mutations along the NS region that could compensate for the reduced fitness caused by the N264D mutation and confer some stability to the TSC-resistant phenotype. Specifically, mutations were detected in NS2 (BVDV R_1_), NS4B (BVDV R_3_), NS5A (BVDV R_2–5_) and NS5B (BVDV R_3_ and R_4_). Particularly, mutation NS5B I261M (BVDV R_3_) has been reported to compensate for the reduced fitness caused by the N264D mutation and to confer resistance to other BVDV NNI [49]–[51]. Additional mutations suggest that the effect of the NS5B N264D mutation on viral fitness could be also counterbalanced by a change in other NS proteins. Because BVDV-TSC^r^ were not replicated under a selective pressure, these additional mutations may have resulted from the genetic drift during the passages and could have also been detected in the wild type virus if it had been passaged in conditions similar to those of BVDV-TSC^r^.

As mentioned above, BVDV R_2_, R_3_, and R_5_ showed a delay in producing CPE. The cp biotypes of BVDV induce cell death by apoptosis [44], [52], triggered by the accumulation of viral double-strand RNA [45], [53] and the continuous expression of NS3, the cleaved form of the nonstructural protein NS2-3 [14], [15]. Although viral RNA production in cp BVDV infections is higher than that in ncp ones, both show similar production of infectious viral particles [18], [53]–[56]. Accordingly, cell cultures infected with BVDV R_2,_ R_3_ and R_5_ showed a decrease in the induction of apoptosis and in the accumulation of viral RNA. However, BVDV R_1–5_ produced higher infectious virus titers than wt BVDV p0, and due to host cell survival, BVDV R_2,_ R_3_ and R_5_ produced viral antigens and infectious viral progeny for at least four additional days.

BVDV NADL cytopathogenicity is determined by an in-frame insertion in the NS2 coding region of a 270-nucleotide cellular mRNA sequence (called cIns), which modulates NS3 production and upregulates RNA replication [18]. As for other cp BVDV strains, the deletion of the inserted host-derived sequences could lead to reversion to the ncp phenotype [54], [57], [58]. In this work, BVDV R_1–5_ showed no deletion (or insertion) in the cIns or in any of the viral sequences. In addition, BVDV R_2,_ R_3_ and R_5_ showed no amino acid changes in NS2 or NS3, whose bond cleavage is required for RNA replication and is linked to BVDV cytopathogenicity [14], [15]. No amino acid changes were detected in NS3-cofactor NS4A or in Npro and Erns, which may play a role in blocking IFN induction and therefore could also affect the viral CPE [5]–[8], [10], [11]. This suggests that the delay of BVDV R_2_, R_3_ and R_5_ in producing CPE may not result from an alteration in the production or function of these viral proteins. On the other hand, BVDV R_3_ showed a mutation in NS4B (E28G). This protein would also play a role in BVDV cytopathogenicity [21], and substitutions for residue NS4B Y15 have been shown to attenuate viral CPE despite NS3 production [55].

Interestingly, most of the additional amino acid changes found in BVDV R_2_ and R_3_ and the only one detected in BVDV R_5_ were in NS5A. BVDV NS5A is a zinc metalloprotein essential for the formation of a functional replicase complex [22], [59], [60] that seems to inhibit TNF-α and double-strand RNA-induced NF-kB activation, which is involved in inflammation, innate immune response, cell survival, and pathogenesis or persistence of BVDV infections [24], [61], [62]. BVDV NS5A is composed of three domains. Mutations were found in domain I (I9M in BVDV R_2_, V177A in BVDV R_5_ and K195E in BVDV R_3_) and domain II (N305Y in BVDV R_2_ and E370K in BVDV R_3_), both critical for RNA replication [22], [63]. Thus, the additional mutations in NS5A could alter its function during viral RNA replication and/or interfere with its interaction with cellular proteins involved in the induction of apoptosis, thus leading to the observed reduction in viral cytopathogenesis.

To our knowledge, this is the first report on the stability and evolution of non-nucleoside polymerase inhibitor-resistant BVDV. The study of the TSC-resistant viruses that showed reduced CPE and prolonged virus production may be an interesting tool to learn more about the virus-cell interaction, viral cytopathogenesis, attenuation and persistence. Further studies and reverse genetic experiments would be needed to identify the specific phenotype of each of the additional mutations observed.
